# Does commitment to rehabilitation influence clinical outcome of total hip resurfacing arthroplasty?

**DOI:** 10.1186/1749-799X-5-20

**Published:** 2010-03-22

**Authors:** David R Marker, Thorsten M Seyler, Anil Bhave, Michael G Zywiel, Michael A Mont

**Affiliations:** 1 Center for Joint Preservation and Replacement, Rubin Institute for Advanced Orthopedics, Sinai Hospital of Baltimore, Baltimore, Maryland, USA; 2 Department of Orthopedic Surgery, Wake Forest University School of Medicine, Winston-Salem, North Carolina, USA; 3 Rehabilitation Services, Rubin Institute for Advanced Orthopedics, Sinai Hospital of Baltimore, Baltimore, Maryland, USA

## Abstract

**Background:**

The purpose of this study was to evaluate whether compliance and rehabilitative efforts were predictors of early clinical outcome of total hip resurfacing arthroplasty.

**Methods:**

A cross-sectional survey was utilized to collect information from 147 resurfacing patients, who were operated on by a single surgeon, regarding their level of commitment to rehabilitation following surgery. Patients were followed for a mean of 52 months (range, 24 to 90 months). Clinical outcomes and functional capabilities were assessed utilizing the Harris hip objective rating system, the SF-12 Health Survey, and an eleven-point satisfaction score. A linear regression analysis was used to determine whether there was any correlation between the rehabilitation commitment scores and any of the outcome measures, and a multivariate regression model was used to control for potentially confounding factors.

**Results:**

Overall, an increased level of commitment to rehabilitation was positively correlated with each of the following outcome measures: SF-12 Mental Component Score, SF-12 Physical Component Score, Harris Hip score, and satisfaction scores. These correlations remained statistically significant in the multivariate regression model.

**Conclusions:**

Patients who were more committed to their therapy after hip resurfacing returned to higher levels of functionality and were more satisfied following their surgery.

## Background

By 2030, the demand for primary total hip arthroplasties is estimated to grow by 174% to 572,000 [1]. The main goal of total hip arthroplasty is to relieve pain and to improve the functional capacity of the patient. Improved functional results lead to a reduced dependence and improved quality of life. Some of the activities of daily living that are affected by arthritis and need to be focused upon after hip arthroplasty include: climbing stairs, shopping, rising out of a chair or bed, housecleaning, washing, and dressing oneself [2]. A large number of these patients will require a major commitment to rehabilitative efforts to attain these functional abilities.

Hip resurfacing arthroplasty has been recommended by some authors as an appropriate treatment modality for certain patients with end-stage degenerative disease of the joint, especially those who are below 65 years of age, have good bone quality, desire to return to a high-activity lifestyles, and have no known metal hypersensitivity [3,4]. Some recent studies have shown that hip resurfacing arthroplasty allows patients to have improved function and reduced pain at short- and mid-term follow-up when compared to standard total hip arthroplasty [5-8]. It has been argued that patient selection as well as intensive rehabilitation following surgery in this subgroup of patients may account for these excellent functional outcomes. For resurfacing, there have been various factors that have been shown to influence successful outcomes following surgery and rehabilitation. For example, it has been suggested that factors such as pre-operative level of activity [9], obesity [10], and gender [11] may affect the outcome. Additionally, patient selection and proper surgical technique is important in avoiding more common complications with this procedure such as femoral neck fracture, and femoral or acetabular component loosening. Although multiple studies have analyzed the effect of rehabilitation on conventional total knee or hip arthroplasty [12,13], there are a limited number of reports that have addressed the influence of patient compliance and the level of commitment to rehabilitation on clinical outcome of hip resurfacing arthroplasty [14,15].

The primary purpose of this study was to assess whether there is any correlation between patient commitment to rehabilitation and their clinical outcomes. The specific questions asked were: 1) Does patient rehabilitation effort correlate with clinical outcome and patient satisfaction?; 2) Do patient characteristics (preoperative diagnosis, gender, body mass index (BMI), age) influence whether a patient is committed to their rehabilitation?; and 3) What additional rehabilitation methods were required for patients who failed initial rehabilitation efforts?

## Methods

A cross-sectional survey was utilized at our hospital to collect information regarding the level of commitment to rehabilitation following hip resurfacing from a series of patients who presented at the authors' center for a scheduled clinical follow-up visit. Completed surveys were received from 147 resurfacing patients (108 men and 39 women). The patients had a mean age of 56 years (range, 20 to 77 years) and a mean body mass index of 28 kg/m^2 ^(range, 18 to 53 kg/m^2^). The men had a mean age of 57 years (range, 37 to 77 years) and a mean body mass index of 29 kg/m^2 ^(range, 21 to 53 kg/m^2^), whereas the women had a mean age of 54 years (range, 20 to 69 years) and a mean body mass index of 26 kg/m^2 ^(range, 18 to 39 kg/m^2^). There were 43 patients who were over 60 years of age and 30 patients who had a body mass index over 30 kg/m^2^. There were 12 patients who had a preoperative diagnosis of osteonecrosis with all other patients having pain and dysfunction associated with advanced primary osteoarthritis. All patients were part of a Food and Drug Administration (FDA)-approved Investigational Device Exemption (IDE) prospective, multi-center, clinical trial.

There were a number of criteria that a patient had to meet to be considered a candidate for metal-on-metal resurfacing hip arthroplasty. Patients were all skeletally mature or at least 18 years old and had to be clinically qualified for a standard total hip arthroplasty based on medical history. Patients who were pregnant, had active human immunodeficiency virus or hepatitis infection, or had a neuromuscular or neurosensory deficiency that might adversely affect gait or weight bearing were not considered for this procedure. Additionally, if a patient had any documented allergy to cobalt, chromium, or molybdenum, they were contraindicated. Patients who had a revision to a standard total hip arthroplasty prior to the final follow-up of this study were not included.

All resurfacing procedures were performed by a single surgeon (MAM) using an antero-lateral approach. The Conserve Plus^® ^hip resurfacing system (Wright Medical Technologies, Arlington, Tennessee) was used for all of the procedures. Standard equipment was used with the femoral head component sizes ranging from 38 to 52 mm. The acetabular components were inserted in a press-fit manner after under-reaming by 1 mm and all femoral components were cemented.

A specific postoperative rehabilitation protocol was used for the IDE study, irrespective of patient age or body mass index. Patients progressed from 20% weightbearing for the first 5 to 6 weeks using crutches or a walker, followed by 50% weightbearing using a cane or crutch in the contralateral hand until 10 weeks, at which time full weightbearing was allowed. Inpatient physical therapy consisted of gait training, low-intensity isometrics, and isotonic exercises of the hip and knee extensors, as well as ankle pumps. Patients were encouraged to maintain hip precautions, which include no flexion past 90°, no adduction past midline, and no hip extension past 0° for ten weeks. Patients were also encouraged to avoid rotation and avoid side-lying active hip abduction. All patients were allowed to weight-bear as tolerated with the aid of a walker or two crutches. Patients continued this program for 6 weeks from the date of surgery. At the end of 6 weeks, patients were given a prescription for outpatient physical therapy. All patients reported similar conventional rehabilitation programs that included progressive resistive exercises of the lower extremity including hip extensors, abductors, knee extensors, and ankle exercises. Patients were encouraged and trained to move from bilateral to unilateral support, and the goal of physical therapy was to achieve ambulation without assistive devices by 10 weeks from the date of surgery.

**Figure 1:**
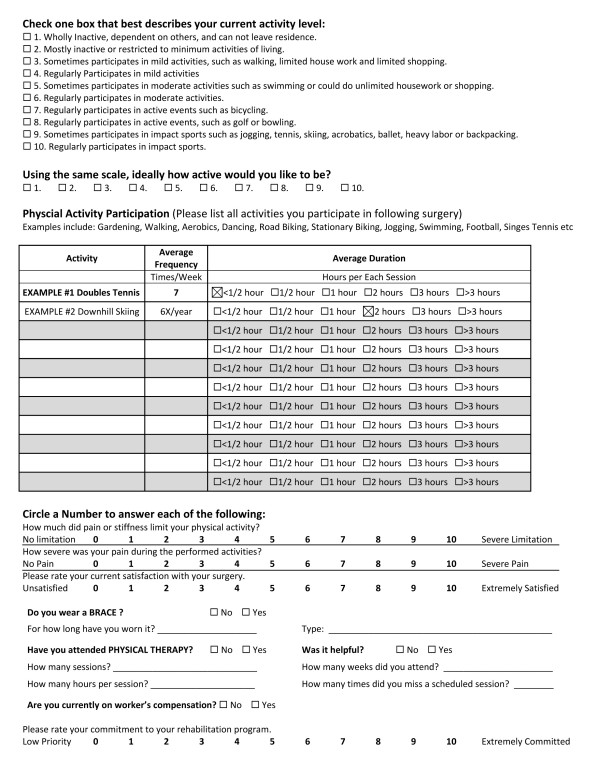
**Activity and rehabilitation questionnaire**. Patients completed this one page questionnaire polling their activity levels and rehabilitation course.

The patients' rehabilitation progress was assessed as part of an expanded version of a previously reported assessment questionnaire (Figure 1) [16]. They were asked to respond to the question; "please rate your commitment to your rehabilitation program," using an eleven-point scale where zero was no effort and poor compliance with the therapy regimen and ten was high effort and 100% compliance. The questionnaire also included a series of post-operative questions related to activity level, competitiveness, and satisfaction.

In addition to the rehabilitation questionnaire, standard clinical outcome measures were collected at a mean follow-up of 52 months (range, 24 to 90 months). Clinical assessments were made prior to surgery and at final follow-up utilizing the Harris hip objective rating system [17]. Functional capability was also assessed at final follow-up using the SF-12 Health Survey and the eleven-point satisfaction score previously described [18]. No surviving patients had any evidence of component loosening or progressive radiolucencies during annual follow-up evaluations which were part of the FDA IDE study protocol. Two patients were revised to a total hip arthroplasty over the follow-up period. One patient underwent revision for a periprosthetic infection eight months following the resurfacing procedure. The second patient was revised at an outside institution for a femoral neck fracture that occurred secondary to a traumatic event four years following the index arthroplasty. Both cases were believed to be unrelated to the rehabilitation procedures.

### Statistical Analysis

All data was collected using a Microsoft Access Database (Microsoft Corporation, Redmond, Washington). Data was exported to SPSS version 13.0 software (SPSS Incorporated, Chicago, Illinois) for statistical analyses. All statistical comparisons were conducted using 95% confidence intervals where a p-value of less than 0.05 was considered significant. For each of the primary questions the following statistics were assessed: 1) Linear regression analysis and Pearson's coefficient were used to determine whether there was any correlation between the rehabilitation commitment scores and any of the outcome measures. A multivariate regression model was used to assess the influence of other factors including age, body mass index, medical comorbidities, diagnoses, and gender; 2) Multivariate regression analysis was used to assess the correlation of various factors with the level of commitment; 3) A Mann-Whitney Rank Sum test was used to compare the outcome scores between various patient populations. These results are shown in Table 1.

**Table 1:** Comparison of outcome scores between various stratified patient groups

	Pre-operative mean HHS (range)	p value	Mean HHS at final follow-up (range)	p value	Mean satisfaction score (range)	p value	Mean SF-12 MCS score (range)	p value	Mean SF-12 PCS score (range)	p value
Men (n = 108) Women (n = 39)	58 (27-78) 51 (30-66)	<0.001	92 (58-100) 92 (69-100)	0.747	8 (0-10) 9 (0-10)	0.008	56 (31-66) 57 (38-64)	0.443	32 (32-60) 51 (26-61)	0.170
										
BMI ≤ 30 (n = 117) BMI >30 (n = 30)	55 (33-70) 57 (27-78)	0.689	89 (76-100) 92 (58-100)	0.048	8 (0-10) 9 (0-10)	0.070	53 (31-61) 57 (38-66)	0.095	51 (32-60) 53 (26-61)	0.068
										
Age ≤ 60 (n = 104) Age >60 (n = 43)	56 (27-78) 55 (30-70)	0.617	92 (69-100) 91 (58-100)	0.519	9 (0-10) 8 (0-10)	0.698	56 (31-66) 57 (39-64)	0.063	53 (26-61) 51 (32-58)	0.524
										
Osteoarthritis (n = 135) Osteonecrosis (n = 12)	56 (27-78) 58 (32-75)	0.319	91 (58-100) 95 (77-100)	0.120	9 (0-10) 10 (7-10)	0.569	56 (31-66) 57 (52-61)	0.816	52 (26-61) 55 (47-57)	0.708

HHS = Harris Hip Score; BMI = Body Mass Index; MCS = Mental Component Score; PCS = Physical Component Score

## Results

Overall, the level of commitment to rehabilitation was shown to predict each of the outcome measures assessed: SF-12 Mental Component Score (r = 0.27; p < 0.001), SF-12 Physical Component Score (r = 0.21; p < 0.001), Harris Hip score (r = 0.23), and satisfaction score(r = 0.35; p < 0.001). These correlations remained statistically significant in the multivariate regression model when controlling for age, body mass index, medical comorbidities, diagnosis, and gender. The overall mean rehabilitation commitment score was 8 points (range, 0 to 10 points). The mean Harris Hip score improved from 56 points (range, 27 to 78 points) prior to surgery to 92 points (range, 58 to 100 points) at final follow-up. At final follow-up, the mean SF-12 mental component score (MCS), physical component score (PCS), and patient satisfaction were 56, 52, and 9 points, respectively.

A comparison of the various clinical outcome measures between patients stratified by various demographic variables (for example, women versus men, high versus low body mass index) revealed a significantly lower mean pre-operative Harris hip score in women compared to men, a significantly lower mean satisfaction level in men compared to women, and a significantly lower mean Harris hip score at final follow-up in non-obese patients (see Table 1). However, these findings should be interpreted with caution as many of these variables may not be truly independent.

**Figure 2:**
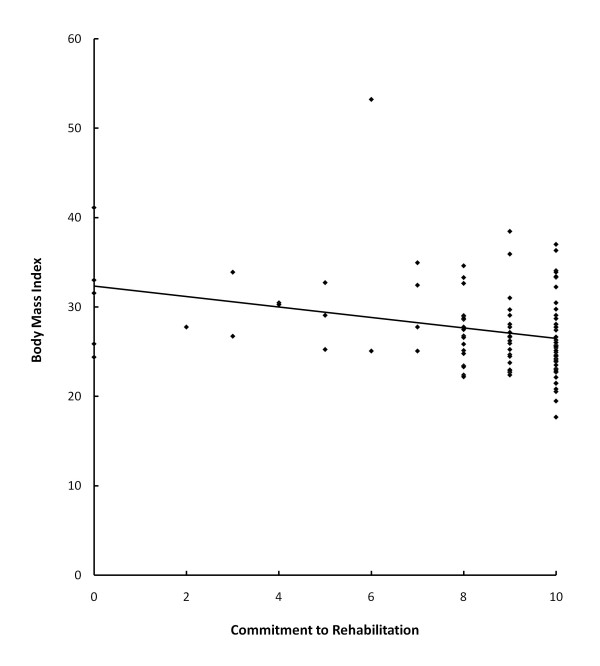
**Body mass index and commitment to rehabilitation**. Plot illustrating the linear correlation between body mass index and commitment to rehabilitation.

The multiple linear regression analysis assessing correlation of various demographic factors and commitment level showed that increasing body mass index had a negative correlation (r = 0.32, p = 0.015; see Figure 2). The results of the analysis of both gender and age were not statistically significant in this model (p = 0.889 and 0.657, respectively).

There were 5 patients who had continued muscle tightness at 3 or more months following surgery despite conventional rehabilitation efforts. Once muscle tightness was identified as an underlying cause of poor functional outcome, these patients were treated with customized therapy at our institution. Their rehabilitation sessions were scheduled 4 or 5 times a week for the first 2 to 3 weeks, and then 3 times a week until functional goals were achieved. Each therapy session included customized stretching consisting of 7 to 10 stretches for each affected muscle. The outpatient regimen included both individual exercises and activities that required the assistance of a family member. This protocol has been previously described as part of a standardized algorithm used at our institution [13].

## Discussion

Total hip resurfacing arthroplasty may allow patients to have comparable function when compared to standard total hip arthroplasty [5-8]. The current levels of patient satisfaction and the timely return to full functional capabilities will potentially be improved with rehabilitation protocols that further develop the coordination between orthopaedic surgeons and other health professionals, as well as with the refinement of surgical techniques, pain management protocols, and appropriate patient expectations.

The limitations of this study include the short-term follow-up mean of 52 months and the still small numbers of patients (n = 147) that make this type of analysis difficult. In addition, following their initial in-patient rehabilitation program, not all patients received physical therapy at the same institution. However, all patients reported similar conventional rehabilitation protocols to make this less of a factor subject to bias.

Efforts have been made to develop standards regarding patient rehabilitation after conventional total hip arthroplasty. Youm et al [19] distributed a questionnaire to the 650 active members of the American Association of Hip and Knee Surgeons to evaluate surgeons' recommendations concerning postoperative rehabilitation and activity restriction. The authors used mean response scores to indicate a recommended standardized postoperative management protocol. Some of these recommendations included the use of an abduction pillow, a high toilet seat, a high chair for 6 weeks, as well as restricted hip flexion for 8 weeks. They also indicated that activities of daily living should be restricted until 5 weeks for driving, 6 weeks for sitting in an office chair, 7 weeks for carrying a brief case, 11 weeks for bending the hips and working on the hands and knees, and 12 weeks for climbing a ladder. Recommended activity levels were dependent on cemented or cementless stems. Nearly all respondents limited weight carrying to 10 pounds at 7 weeks for cemented stems and 8 weeks for cementless stems. While standardized rehabilitation techniques provide excellent results in most patients, the results of the present study suggest that there are some patients who may require additional customized protocols, especially younger patients. In addition, certain patients may need less rehabilitation.

In addition to establishing standards for the participation in functional activities and rehabilitation protocols, the use of a comprehensive, multidisciplinary, inpatient rehabilitation regimen has been shown by Dohnke et al [20] to be important in providing optimal outcomes after total hip arthroplasty. Their study evaluated the clinical outcome of 1,065 total hip arthroplasty patients for whom a coordinated multidisciplinary approach was followed. The inpatient rehabilitation began approximately 3 weeks (mean 22 days) after surgery, and the mean length of stay was approximately 23 days. Significant improvements in disability, pain, depressive symptoms, and ability to function independently were made postoperatively from the time of admission to discharge from the inpatient rehabilitation program.

While the present study suggests that current rehabilitation protocols for hip resurfacing patients yield satisfactory results, it remains unclear whether these programs are optimal. The protocols were originally designed for total hip arthroplasty patients who often are older and less active than many resurfacing patients. In the present study, there were three patients who discontinued rehabilitation after reaching all functional goals by 6 weeks post-operatively. Based on their excellent results and accelerated progress, they were cleared by their physical therapist (AB) and surgeon (MAM) from any additional prescribed rehabilitation. These results were similar to those reported by Crow et al. who found that a multimodal treatment approach allowed a 43 year-old man to return to sports activity following bilateral resurfacing [21]. Their rehabilitation approach focused on joint mobilization and the patient achieved approximately 90 degrees of hip flexion and 10 degrees of lateral rotation bilaterally by 3 months postoperatively. In another study, Newman et al. also suggested that new rehabilitation standards may need to be adapted for resurfacing patients [15]. They assessed the outcomes of 126 hip resurfacing patients and reported excellent return to function following resurfacing with a mean Oxford Hip Score of 15 points and UCLA Activity Score of 7 points. However, they reported that approximately 1 out of 4 of the patients reported persistent pain with decreased strength and a reduced hip flexion at a mean of 95 degrees (+/- 13 degrees). They concluded that the suboptimal recovery for some of their cohort may have been attributed to the rehabilitation protocols that were originally developed for standard total hip arthroplasty patients and not for their resurfacing arthroplasty counterparts.

Based partly on the results of this study, we currently we allow considerable variation from the previously described protocol for patients who are treated with a hip resurfacing arthroplasty, with progression based on the ability to achieve certain functional goals, rather than using only time since the index arthroplasty, which has most often been used in the past. Thus, some patients can be treated in an individual manner based on their ability to achieve certain functional milestones. Our current rehabilitation goal by five weeks following surgery is for the patient be able to ambulate pain free using single point cane in the opposite hand, go up and down the a flight of stairs, flex their hip to 90 degrees, and abduct to 30 degrees. We avoid strengthening exercises of the hip that are associated with pain, and specifically avoid side lying hip abduction strengthening early because of our anterolateral surgical approach. If patients achieve these well-defined goals earlier than 5 weeks we recommend faster progression to full range of motion, including rotation. We also progress patients to weight bearing as tolerated without the use of an assistive device, and place patients on progressive resistive exercises to improve hip abductor and extensor strength as long as resistive exercise does not cause pain. Accelerated, rather than time based, rehabilitation performed in this fashion may reduce the total time spent in rehabilitation for a number of patients. This preliminary study suggests that in general, a major commitment to rehabilitation should be made by patients to achieve the best clinical outcomes. In addition, patients who remain stiff or have difficulty progressing may require additional, tailored rehabilitation regimens. Conversely, patients who rapidly regain excellent function and a high activity level following surgery may be able to avoid further rehabilitation once certain goals are met. However, further investigation and multi-center studies need to be performed to confirm and refine these conclusions.

Based on the results of the current study, we suggest that increased body mass index may have a negative correlation with patient commitment to rehabilitation. Similar results were reported by Vincent et al. who examined whether obesity affected inpatient rehabilitation outcomes after total hip arthroplasty [22]. In their study, all patients completed an interdisciplinary inpatient rehabilitation program after surgery and were evaluated using functional independence measure scores, length of stay, efficiency scores (functional independence measure scores/length of stay), hospital charges, and discharge disposition location. Although functional independence measure scores improved from admission (mean of 25 points) to discharge (mean of 29.5 points) in all groups, the efficiency scores, length of stay functional independence measure scores, length of stay, and total charges were curvilinearly related to body mass index. They concluded that while elevated body mass index does not prevent functional gains in total hip arthroplasty patients during inpatient rehabilitation, increasing body mass index does influence efficiency, length of stay, and hospital charges in a negative manner. Furthermore, severely obese patients can achieve physical improvements, but at a lower efficiency and greater cost.

The use of a comprehensive activity and rehabilitation tool such as the one reported in the present study may allow surgeons to predict the postoperative recovery course for patients for hip resurfacing as well as other arthroplasty treatments, and allow for a tailoring of rehabilitation treatments. Additionally, it may assist surgeons in providing guidance regarding which treatment modality may be most appropriate for a given patient. Further study is necessary to better define these potential benefits.

## Conclusions

The results of this study suggest that the level of commitment to rehabilitation influences outcomes with hip resurfacing, as we found that patients in our cohort who were more committed to their therapy returned to higher levels of functionality and satisfaction. The excellent early clinical outcomes following successful hip resurfacing in our cohort are similar to the results of other studies that have assessed modern hip resurfacing prostheses. We suggest that the importance of rehabilitation compliance should be stressed to resurfacing patients following surgery so that they can achieve maximal functional improvement and a healthier lifestyle.

## Competing interests

External financial support was received specifically in support of the database used in this study from Wright Medical Technologies (Arlington, Tennessee). MAM is a consultant for Stryker Orthopaedics and Wright Medical Technologies.

None of the other authors have any financial or non-financial competing interests to disclose.

## Authors' contributions

DRM, AB, TMS, MAM, MGZ designed the study. DRM, MGZ, TMS collected the data. DRM, MGZ, TMS analyzed the data. DRM, MGZ, AB prepared the manuscript. AB, MGZ, TM, DRM, MAM ensured the accuracy of the data and analysis. All authors have read and approved the final manuscript.
